# Investigating heart rate variability measures during pregnancy as predictors of postpartum depression and anxiety: an exploratory study

**DOI:** 10.1038/s41398-024-02909-9

**Published:** 2024-05-14

**Authors:** Allison Eriksson, Mary Claire Kimmel, Tomas Furmark, Anna Wikman, Marcus Grueschow, Alkistis Skalkidou, Andreas Frick, Emma Fransson

**Affiliations:** 1 Department of Women’s and Children’s Health, Uppsala University, Uppsala, Sweden; 2 Women’s Mental Health during the Reproductive Lifespan – WOMHER, Uppsala University, Uppsala, Sweden; 3 Department of Psychiatry, University of North Carolina, Chapel Hill, NC USA; 4 Department of Psychology, Uppsala University, Uppsala, Sweden; 5 Zurich Center for Neuroeconomics (ZNE), Department of Economics, University of Zurich, Zurich, Switzerland; 6 Department of Medical Sciences, Psychiatry, Uppsala University, Uppsala, Sweden; 7 Department of Microbiology, Tumor and Cell Biology, Karolinska Institute, Stockholm, Sweden

**Keywords:** Predictive markers, Neuroscience, Physiology

## Abstract

Perinatal affective disorders are common, but standard screening measures reliant on subjective self-reports might not be sufficient to identify pregnant women at-risk for developing postpartum depression and anxiety. Lower heart rate variability (HRV) has been shown to be associated with affective disorders. The current exploratory study aimed to evaluate the predictive utility of late pregnancy HRV measurements of postpartum affective symptoms. A subset of participants from the BASIC study (Uppsala, Sweden) took part in a sub-study at pregnancy week 38 where HRV was measured before and after a mild stressor (*n* = 122). Outcome measures were 6-week postpartum depression and anxiety symptoms as quantified by the Edinburgh Postnatal Depression Scale (EPDS) and the Beck Anxiety Inventory (BAI). In total, 112 women were included in a depression outcome analysis and 106 women were included in an anxiety outcome analysis. Group comparisons indicated that lower pregnancy HRV was associated with depressive or anxious symptomatology at 6 weeks postpartum. Elastic net logistic regression analyses indicated that HRV indices alone were not predictive of postpartum depression or anxiety outcomes, but HRV indices were selected as predictors in a combined model with background and pregnancy variables. ROC curves for the combined models gave an area under the curve (AUC) of 0.93 for the depression outcome and an AUC of 0.83 for the anxiety outcome. HRV indices predictive of postpartum depression generally differed from those predictive of postpartum anxiety. HRV indices did not significantly improve prediction models comprised of psychological measures only in women with pregnancy depression or anxiety.

## Background

Physiological and emotional challenges inherent to pregnancy and childbirth, such as substantial changes in hormone levels, alterations in the cardiovascular and immune systems [1, 2], and concerns about impending parenthood, can reveal psychiatric vulnerability in many women, potentially culminating in perinatal affective disorders [3–7]. An estimated 10–20% of women suffer from postpartum depression (PPD [6, 8]); and 20% of women meet the criteria for at least one anxiety disorder during pregnancy and the postpartum period [7]. Previous studies have shown that postpartum women who suffer from untreated depression and anxiety are more likely to engage in substance/alcohol abuse [9], and have an increased risk of suicide [10–12]. Further, untreated depression and anxiety can negatively affect maternal bonding, which is fundamental to the development of secure child-mother attachment and healthy child development [13–15]. Children of mothers with postpartum depression and anxiety are at an increased risk of becoming malnourished, having poor growth rates, having sleep disturbances, and contracting certain illnesses [16]. Emotional problems, such as difficulty socializing and internalizing behavior [10], behavioral problems [17], and physiologic markers such as increased cortisol levels [18], are more prevalent among children of mothers with untreated depression and/or anxiety.

There are interventions with proven efficacy to prevent postpartum mental ill-health [19]; however, these interventions are primarily effective among high-risk groups [20], thereby rendering early identification of women at higher risk for postpartum depression and anxiety crucial. Research shows that only a small proportion of women with depressive symptoms are identified and adequately treated despite routine screening [21–24]. Further, a study from the UK revealed that only about 30% of peripartum women suffering from mental health issues were open about their symptoms [25], which is particularly problematic as current screening procedures rely entirely on traditional screening methods comprised of subjective self-reports [26]. Considering the gravity of postpartum depression and anxiety on maternal and infant outcomes, it is imperative that novel, objective measures aimed at predicting women who are at high risk for postpartum depression and anxiety are explored and implemented.

### Heart rate variability and affective disorders

Research has examined ways in which the body exhibits quantifiable markers of affect regulation. A well-functioning system is marked by its flexibility to meet ever-changing environmental demands [27, 28]. Previous studies have asserted that affective disorders, such as postpartum depression and anxiety, may stem partly from maladaptive regulatory processes within the two branches of the autonomic nervous system—the sympathetic and parasympathetic nervous systems [27]. In particular, recovery after stress called the adaptive response, is thought to be dysregulated in affective disorders [29]. One indirect physiological measure of parasympathetic and sympathetic activity is heart rate variability (HRV), the non-invasive measurement of beat-to-beat changes in heart rate [30]. HRV is mediated by the parasympathetic nervous system, or vagal nerve, which slows the heart rate, and the sympathetic nervous system, which accelerates heart rate [31, 32]. Regulation of heart rate is intrinsically coupled with the body’s ability to react, and subsequently adapt, to emotional and environmental stimuli; thereby making HRV a proxy for stress reactivity within the autonomic nervous system [8, 33, 34]. Higher HRV is indicative of a healthy system capable of responding to stimuli while inhibiting excessive or unnecessary responses [27, 35]. A dysregulated system, represented by lower HRV, is considered unresponsive to environmental stimuli; therefore, unable to select appropriate responses or inhibit inappropriate responses [27, 36]. It has been suggested affective and cardiac disorders could interact in a ‘downward spiral’, in which they reinforce each other [37, 38], and it could be reasonable to hypothesize that HRV could be indicative of both heart and brain alterations at an early stage. A relatively new area of research investigating the brain-heart interplay has linked subclinical depression (dysphoria) to alterations of the functional central-autonomic control of the heart [39].

Previous studies have shown lower HRV to be associated with various forms of pathology, including affective disorders, hypertension, and coronary heart disease [27, 32, 33, 35, 40]. In a non-pregnant sample, HRV indices measured during and after a mild stressor exhibited a screening sensitivity of 80% for Major Depressive Disorder; higher than using the subjective patient-reported screening method alone [41]. Relatively higher HRV measures have been associated with a lower likelihood of future depressive symptoms in a population-based study [42]. In addition, anxiety-related disorders have been studied in connection to HRV measures, as well. Zhang and colleagues [43] reported a relationship between non-pregnant patients diagnosed with panic disorder and changes in HRV indices. A meta-analysis focused on HRV measures in relation to various anxiety-related disorders, such as panic disorder, post-traumatic stress disorder (PTSD), social anxiety disorder, and generalized anxiety disorder (GAD), indicated that anxiety disorders are generally characterized by lower HRV [44]. Interventions for improving HRV via HRV biofeedback (HRVB) [35, 45] have been shown to improve depressive and anxious symptomatology. A recent systematic review concluded that HRVB significantly improved symptoms of stress-related disorders, as well as symptoms of depression [46].

Studies examining HRV during pregnancy and the postpartum period have been scarce and have primarily shown associations between altered autonomic activity and symptoms of depression and stress in pregnant women [47, 48]. Other studies have reported significantly altered HRV indices among pregnant women with current and past anxiety disorders and symptoms of current anxiety [8, 49, 50]. A recent study by Singh Solorzano and colleagues [35] demonstrated that reduced parasympathetic activity during pregnancy, measured via one HRV index, was associated with higher depressive symptoms in the postpartum period.

The aim of the current study was to conduct an exploratory analysis to investigate if proxies of the stress and adaptive responses, operationalized as a variety of late pregnancy HRV indices measured before and after a stressor, could predict postpartum depression and anxiety. More specifically, we hypothesized that lower HRV parameters in gestational week 38 would predict the presence of symptoms of depression and anxiety at 6-weeks postpartum.

## Materials and methods

### Subjects and procedure

A total of 5492 participants were enrolled in the Biology, Affect, Stress, Imaging, and Cognition (BASIC) study beginning at gestational weeks 16–18 in Uppsala, Sweden. The BASIC study was conducted between 2009 and 2018 [51] and represented 6478 pregnancies. Exclusion criteria consisted of inadequate understanding of Swedish, age under 18 years, protected identity, bloodborne illness, and a non-viable pregnancy diagnosed by routine ultrasound. A subgroup of participants from the BASIC cohort took part in a sub-study related to HRV measurement at gestational week 38 (mean days before birth = 11, SD = 5.9). Participants who indicated elevated distress on the EPDS at gestational week 32 were oversampled. A sample of 122 women representing 122 pregnancies participated in HRV measurements between 2014 and 2018. The participation rate in the sub-studies within the whole BASIC cohort study was 48.8% for pregnancy test sessions [51]. On the day of the visit, participants completed neuropsychiatric questionnaires prior to HRV measurement. HRV analysis was performed using photoplethysmography (PPG), a method of measuring pulse-rate variability via an electrode placed on each index finger, which has been shown to accurately measure HRV [52, 53]. All HRV indices were obtained using a PPG transducer (models PPG stress flow, provided by BioTekna, Italy) at two time-points; an initial 5-min HRV segment was recorded (referred to as “baseline”). Then the participants underwent a working memory task as a slight stressor, the Wechsler Digit Span Test (DST [54]); A second 5-min segment of HRV collection followed (referred to as “after stressor”). These two time-points are thought to represent different aspects of the stress response. Participants with incomplete HRV data or missing data in the depression or anxiety outcome measures were excluded from the final analysis resulting in a sample size of *n* = 112 for the depression outcome and *n* = 106 for the anxiety outcome. Sample sizes were based on comparable studies and deemed to be sufficient for our study protocol.

### Ethical considerations

This project followed the ethical guidelines set out by the Swedish Ethical Review Authority and GDPR requirements. Ethical permits have been obtained for the BASIC study (EPN Uppsala 2009/171 with amendment 2009/171/2 from 2014.). Written informed consent was obtained from all participants to participate in the BASIC study, as well as prior to participation in the sub-study. Data related to childbirth was retrieved from medical records.

### Background, pregnancy, and psychological self-report measures

Participants completed web-based questionnaires, as well as psychological measures of anxiety and depressive symptoms at gestational weeks 17, 32, and 38, and postpartum six weeks. The web surveys included the Edinburgh Postnatal Depression Scale (EPDS) for assessment of depressive symptoms [55], and the Beck Anxiety Inventory BAI [56]; for assessment of anxiety symptoms at gestational week 32. The 14-item Resilience Scale (RS-14) was also administered at gestational week 32 [57, 58] to assess the influence of resilience level on our outcome variables. Demographic data included information related to place of birth, maternal age, parity, and education level. We also evaluated participants’ body mass index (BMI) and history of depression and/or anxiety, given their known association with changes in HRV parameters [40, 59]. A binary, composite variable named “Physical illness” was statistically created to encompass participants who indicated the presence or absence of any physical illness prior to pregnancy. Physical illnesses included hypertension, asthma, preeclampsia, cardiovascular disease, thrombosis, endocrine disease, and anemia. Data acquired during pregnancy included variables related to sleeping habits, fear of delivery, and use of the antidepressant medication class, selective serotonin reuptake inhibitors (SSRIs).

### Psychological outcome measures

Our outcome measures were depression and anxiety symptoms at six weeks postpartum. Participants with a total score on the EPDS between 0 and 10 at 6 weeks postpartum were classified as “Non-depressed”, while participants with an EPDS score between 11 and 30 were classified as “Depressed”. For anxiety measures, participants with a total BAI score between 0 and 15 at 6 weeks postpartum were classified as “Non-anxious”, while participants with a total BAI score of 16 or above were classified as “Anxious”.

### HRV feature analysis

HRV is most commonly measured in the time-domain and frequency-domain [30]. Time-domain measures calculated in this study include both the standard deviation of normal-normal intervals (SDNN), which reflects both sympathetic and parasympathetic function, and the root mean square of successive differences (RMSSD), which is sensitive to parasympathetic variation [40]. Frequency-domain measures include: power (LF; 0.04 ∼ 0.15 Hz); high-frequency power (HF; 0.15 ∼ 0.4 Hz); total power; and LF/HF ratio. LF power is regulated by the sympathetic and parasympathetic nervous systems, while HF power is regulated by parasympathetic activity. LF/HF ratio is, often, presumed to reflect a balance between sympathetic and parasympathetic activities [30, 40, 59]. Total power is a measurement of all the frequency domain measurements, reflecting a number of different components of the ANS including parasympathetic and sympathetic contribution [30] Total power has been shown in previous studies to differ between healthy individuals and individuals with anxiety and stress-related disorders [8, 43, 60]. All frequency-domain HRV indices were log-transformed to provide a more normal distribution [61].

### Statistical analysis

Statistical analysis was performed using R programming language [62] through RStudio version 2022.07.2 [63] and results were considered significant at *p* < 0.05. HRV indices were computed by BioTekna (Italy). Bivariate analyses consisted of conducting chi-square tests of independence for group differences in background and pregnancy variables in relation to the development of symptoms of postpartum depression and anxiety at 6-weeks postpartum. An independent samples *t*-test was conducted for group differences in BMI. Independent sample *t*-tests were also conducted for group differences based on postpartum depression and anxiety outcomes and all HRV indices with the exception of baseline and after stressor LFHF ratio and RMSSD indices; these HRV indices were found to not be normally distributed and non-parametric Wilcoxon rank sum tests were conducted accordingly. Finally, elastic net logistic regression [64], described in detail in the following section, was conducted to test our hypothesis that late pregnancy HRV measurements are predictive of postpartum development of symptoms of depression and anxiety.

#### Imputation and model selection

In the development of our predictive model, imputation of missing data within our self-report variables was conducted to avoid data loss. Self-report variables consisted of background variables (place of birth (Scandinavia vs other), maternal age (in years), parity (nulliparous vs multiparous), education level (university vs lower), BMI, depression history (yes vs no)), pregnancy variables (sleeping habits (less than 6 h/6 – 8 h/more than 8 h), fear of delivery (yes vs no), SSRI use (yes vs no), Physical illness (yes if any of the conditions hypertension, asthma, preeclampsia, cardiovascular disease, thrombosis, endocrine disease, or anemia vs no), and clinical measures collected at gestational week 32 (EPDS, BAI, RS-14)). Prior to imputation, careful inspection of patterns of missingness within the self-report variables was examined with the R package “nanair” [65] and xmissingness was determined to be quite low (2.8%). Data were found to fulfill the assumption of missing at random and, therefore, deemed to be suitable for multiple imputation. Imputation was executed via multivariate imputation by chained equations (MICE) using the “mice” package in R [66] and the univariate imputation method employed was predictive mean matching.

Elastic net logistic regression using R package “glmnet” [67] was used to generate receiver operating characteristic (ROC) curves using R package “pROC” [68]. Elastic net regression is a regulation and variable selection method that utilizes a penalty for model complexity and is particularly well-suited to data analysis involving many predictor variables that are highly correlated [64]. A combination of self-report measures and HRV indices (combined models) were included in the elastic net logistic regression analyses to find the best predictor variables for postpartum symptoms of depression or anxiety [69]. All variables included in the elastic net regression analyses were Z-score transformed to create a standardized distribution and allow comparison between the measures [70]. For the depression outcome, data were randomly split into two groups; 80% of participants created one group that was used for training the prediction model, while the remaining 20% of participants created a second group that was used to test the accuracy of the model. Since the number of women who developed postpartum symptoms of anxiety was small, the groups were divided into 50% training and 50% test [71]. A tenfold cross-validation was conducted on the training data to reduce overfitting and improve prediction accuracy [72], and optimal values of the regularization parameters alpha (α) and lambda (λ) were obtained for best model performance [73].

Elastic net logistic regression was conducted using optimal α and λ parameters to create a prediction model. ROC analyses were then conducted to determine the classification accuracy of the model on the test data via the area under the curve (AUC), which is a graphical representation of the sensitivity, or true positive rate, versus the specificity, or true negative rate, of a model. A model with perfect prediction accuracy would have a ROC curve with an AUC = 1.0, while a ROC curve with an AUC = 0.5 would indicate the model’s classification accuracy corresponds to random chance (See Zou et al. [69] for a more detailed description of ROC analysis). Additionally, two comparison models for each outcome were generated using precisely the same method described above, but (1) excluding HRV variables and (2) including only HRV variables. By comparing models with and without HRV indices we can quantify the precise predictive utility of HRV indices above and beyond all other variables.

## Results

### Background, pregnancy, and psychological self-report measures

Twenty-six (23.2%) of the 112 women with depression outcome scores reported symptoms of postpartum depression (four de-novo cases, 3.6%). Twelve (11.3%) of the 106 women with anxiety outcome scores reported symptoms of postpartum anxiety (one de-novo case, 0.9%). Of women with symptoms of postpartum depression or anxiety, 17 women reported symptoms of only depression, 3 women reported symptoms of only anxiety, and 9 women reported symptoms of both depression and anxiety. Results showed that there were no significant differences between groups in background variables, while group differences were present in pregnancy and psychological variables (Table 1).

**Table 1:** Background, pregnancy, and psychological variables by depression and anxiety outcomes for participants at 6-weeks postpartum.

Background	Non-depressed *n* (%)	Depressed *n* (%)	Non-anxious *n* (%)	Anxious *n* (%)	*P*-value	
					Depressed	Anxious
**Place of birth**						
Scandinavia	78 (95.1)	23 (92)	86 (95.5)	10 (83.4)		
Other	4 (4.9)	2 (8)	4 (4.5)	2 (16.6)	0.552	0.091
**Maternal age**						
21–29	28 (32.6)	9 (36)	32 (34)	2 (18.2)		
30–34	33 (38.3)	13 (52)	39 (41.5)	6 (54.5)		
35–42	25 (29.1)	3 (12)	23 (24.5)	3 (27.3)	0.204	0.552
**Education**						
University	64 (76.2)	22 (88)	72 (78.3)	12 (100)		
Less	20 (23.8)	3 (12)	20 (21.7)	0 (0)	0.336	0.072
**Parity**						
0	46 (59.7)	13 (54.2)	48 (57.1)	6 (54.5)		
1 or more	31 (40.3)	11 (45.8)	36 (42.9)	5 (45.5)	0.628	0.870
**BMI**						
Mean *(SD)*	28.2 *(3.9)*	28.6 *(4.4)*	28.1 (4.3)	29.4 *(3.7)*	0.610	0.379
**Physical illness**						
0.911	62 (81.6)	19 (82.6)	66 (80.5)	10 (90.9)		
0.911	14 (18.4)	4 (16.7)	16 (19.5)	1 (9.1)	0.911	0.401
**Previous depression**						
No	26 (31.3)	4 (14)	66 (80.5)	10 (90.9)		
Yes	57 (68.7)	21 (86)	16 (19.5)	1 (9.1)	0.134	0.401
**Pregnancy**	**Non-depressed** ***n*** **(%)**	**Depressed** ***n*** **(%)**	**Non-anxious** ***n*** **(%)**	**Anxious** ***n*** **(%)**	***P*-value**	
					**Depressed**	**Anxious**
**Sleep week 32**						
Less than 6 h	6 (6.9)	4 (15.4)	8 (8.5)	2 (16.7)		
6–8 h	62 (72.1)	13 (50)	61 (64.8)	9 (75)		
More than 8 h	18 (20.9)	9 (34.6)	25 (26.6)	1 (8.3)	0.101	0.307
**Fear of delivery week 32**						
No	61 (70.9)	13 (50)	67 (71.3)	3 (25)		
Yes	25 (29.1)	13 (50)	27 (28.7)	9 (75)	**0.048**	**0.001**
**SSRI week 17/32**						
No	74 (96.1)	22 (88)	81 (96.4)	9 (75)		
Yes	3 (3.9)	3 (12)	3 (3.6)	3 (25)	0.135	**0.004**
**Psychological**	**Non-depressed** ***n*** **(%)**	**Depressed** ***n*** **(%)**	**Non-anxious** ***n*** **(%)**	**Anxious** ***n*** **(%)**	***P*-value**	
					**Depressed**	**Anxious**
**Resilience week 32**						
Very low to low	18 (23.1)	11 (61.1)	30 (33.3)	8 (72.7)		
Moderate to high	60 (76.9)	7 (38.9)	60 (66.7)	3 (27.3)	**0.001**	**0.011**
**Depressive symptoms week 32**						
0–10	64 (74.4)	4 (15.4)	64 (68.1)	1 (8.3)		
11–30	22 (25.6)	22 (84.6)	30 (31.9)	11 (91.6)	**<0.001**	**<0.001**
**Anxiety symptoms week 32**						
Minimal/Mild	57 (67.1)	9 (33.3)	60 (65.9)	3 (25)		
Moderate/Severe	28 (32.9)	18 (66.7)	31 (34.1)	9 (75)	**0.002**	**0.006**

*BMI* Body Mass Index, *Physical illness* hypertension, asthma, preeclampsia, cardiovascular disease, thrombosis, endocrine disease, and anemia. Bold values indicate *p* ≤ 0.05.

### HRV indices and postpartum depression and anxiety

Results of tests for mean differences in week 38 HRV indices between non-depressed/non-anxious participants and depressed/anxious participants at 6-weeks postpartum are shown in Table 2. We found that certain HRV indices were significantly lower depending on depressed/anxious outcome versus non-depressed/non-anxious outcome. The depression outcome was significantly associated with lower RMSSD baseline and SDNN after stressor. There was a trend toward significant results for baseline indices LF power, Total power, and SDNN as well as for after stressor indices HF power and Total power. The anxiety outcome was significantly associated with lower HRV in two after stressor measurements, Total power and SDNN. There was also a trend towards significant results in the after stressor measurements for RMSSD and LF/HF ratio.

**Table 2:** Independent samples *t*-tests for differences in heart rate variability (HRV) indices in non-depressed/non-anxious and depressed/anxious women at 6-weeks postpartum.

HRV Category	Type of scale	Non-depressed/anxious		Depressed/Anxious		*p*-value
		*n*	*M* *(SD)*	*n*	*M* *(SD)*	
**RMSSD baseline**^**+**^	**Depression**	**86**	**32.7** **(27)**	**26**	**23.0** **(11)**	**0.049**
	Anxiety	94	29.2 (15.8)	12	36.1 (55.3)	0.292
**RMSSD after stressor**^**+**^	Depression	86	33.7 (19.2)	26	28.1 (13.9)	0.214
	Anxiety	94	32.8 (17.6)	12	24.8 (14.1)	0.093
**SDNN baseline**	Depression	86	56.2 (22)	26	47.2 (21)	0.010
	Anxiety	95	53.9 (20.1)	14	51.8 (32.6)	0.975
**SDNN after stressor**	**Depression**	**86**	**55.7** **(21)**	**26**	**46.5** **(17)**	**0.032**
	**Anxiety**	**94**	**54.3** **(20.6)**	**12**	**41.5** **(13.4)**	**0.009**
**HF power baseline**	**Depression**	**86**	**5.93** **(1.2)**	**26**	**5.25** **(1.3)**	**0.027**
	Anxiety	95	5.8 (1.6)	14	5.46 (1.6)	0.499
**HF power after stressor**	Depression	86	6.09 (1.1)	26	5.64 (1.1)	0.085
	Anxiety	94	6.04 (1.0)	12	5.44 (1.3)	0.141
**LF power baseline**	Depression	86	6.03 (.8)	26	5.6 (1.0)	0.064
	Anxiety	95	5.95 (.86)	14	5.86 (1.1)	0.794
**LF power after stressor**	Depression	86	6.1 (.74)	26	5.9 (.92)	0.436
	Anxiety	94	6.07 (.76)	12	5.9 (.82)	0.500
**Total power baseline**	Depression	86	7.89 (.7)	26	7.55 (.8)	0.073
	Anxiety	94	7.83 (.7)	12	7.64 (.8)	0.473
**Total power after stressor**	Depression	86	7.88 (.7)	26	7.53 (0.7)	0.058
	**Anxiety**	**94**	**7.83 (.7)**	**12**	**7.35 (.65)**	0.**034**
**LF/HF ratio baseline**^**+**^	Depression	86	1.6 (1.6)	26	1.7 (1.5)	0.194
	Anxiety	94	1.62 (1.6)	12	1.88 (1.7)	0.325
**LF/HF ratio after stressor**^**+**^	Depression	86	1.45 (1.3)	26	1.97 (2.2)	0.161
	Anxiety	94	1.52 (1.6)	12	2.09 (1.7)	0.088

*Note* ^**+**^Analysis performed with Wilcoxon signed rank non-parametric test; “Baseline” refers to the first 5-min HRV recording; “after stressor” refers to the second 5-min HRV recording after DST. Bold values indicate *p* ≤ 0.05.

*RMSSD* root mean square of successive differences, *SDNN* standard deviation of normal-normal intervals, *HF* high frequency, *LF* low frequency.

### ROC analysis with elastic net logistic regression model summary for depression outcome

**Fig. 1:**
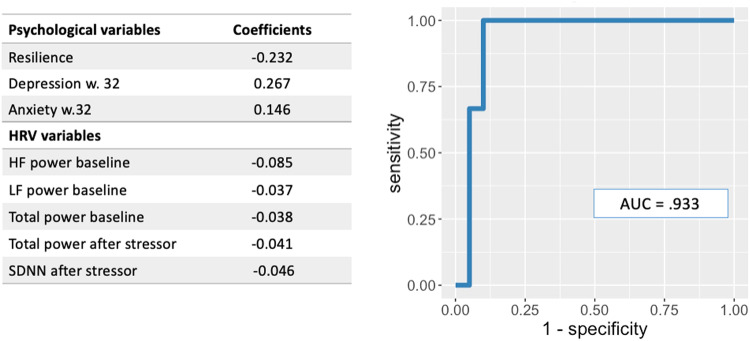
AUC and significant predictors from ROC analysis with elastic net logistic regression for model with depression outcome. *Note*. Depression outcome based on EPDS score ≥ 11; Resilience based on RS-14; Depression w. 32 = total EPDS; Anxiety w.32 = total BAI; HF high frequency, SDNN standard deviation of normal-normal intervals, Model parameters: α = 0.25 and λ = 0.25.

In the elastic net logistic regression analysis for depression outcome (non-depressed v. depressed participants at 6-weeks postpartum), model parameters were α = 0.25 and λ = 0.25. ROC analysis revealed a total of 7 predictor variables for depression symptoms at 6-weeks postpartum; these included 3 psychological variables (lower resilience, higher depression symptoms, higher anxiety symptoms reported at 32 weeks of pregnancy) and 5 HRV variables at 38 weeks of pregnancy (HF power baseline, LF power baseline, Total power baseline, Total power after stressor, SDNN after stressor). The AUC for this model was 0.933 (Fig. 1).

### ROC analysis with elastic net logistic regression model summary for anxiety outcome

**Fig. 2:**
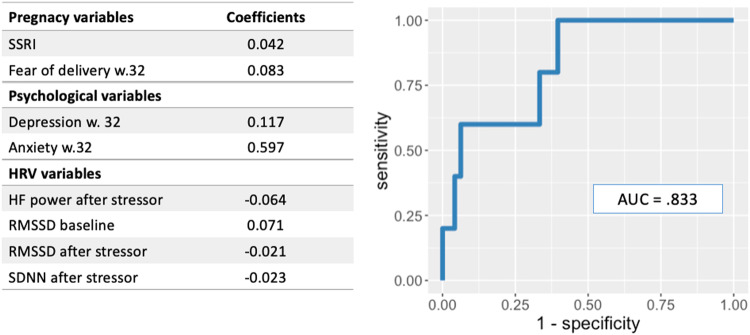
AUC and significant predictors from ROC analysis with elastic net logistic regression for model with anxiety outcome. *Note*. Anxiety outcome based on postpartum BAI scores ≥ 16; Anxiety w. 32 = total BAI score pregnancy week 32; Depression w. 32 = total EPDS score pregnancy week 32; HF high frequency, RMSSD root mean square of successive differences, SDNN standard deviation of normal-normal intervals, Model parameters: α = 0.45 and λ = 0.15.

In the elastic net logistic regression analysis for anxiety outcome (non-anxious v. anxious participants at 6-weeks postpartum), model parameters were α = 0.45 and λ = 0.15. ROC analysis revealed a total of 8 predictor variables for anxiety symptoms at 6-weeks postpartum; these included 2 pregnancy variables (SSRI use, greater fear of delivery), 2 psychological variables (higher depression symptoms, higher anxiety symptoms reported at 32 weeks of pregnancy), and 4 HRV variables at 38 weeks of pregnancy (HF power after stressor, RMSSD baseline, RMSSD after stressor, SDNN after stressor). With the exception of SDNN after stressor, HRV indices predictive for the anxiety outcome were distinct from those predictive for the depression outcome. The AUC for this model was 0.833 (Fig. 2).

### Comparison models excluding HRV indices

Comparison models with depression and anxiety outcomes excluding HRV indices were created using elastic net logistic regression analysis. The depression outcome model gave an AUC = 0.916 (Supplementary Fig. 1). The anxiety outcome model excluding HRV indices gave an AUC = 0.806 (Supplementary Fig. 2) Models including only HRV variables were not predictive (AUC for depression =0.587, for anxiety 0.5)

## Discussion

This study investigated if HRV indices measured before and after a working memory task that served as a mild stressor during gestational week 38 could predict the presence of symptoms of depression and anxiety at 6-weeks postpartum. Certain HRV indices were significantly lower in women who reported postpartum depression and anxiety. However, we found that HRV indices alone were not predictive of postpartum depression or anxiety, and did not significantly improve the predictive power of models comprised of psychological scales in women with pregnancy depression and anxiety. Still, it is of interest that HRV indices selected as significant predictors of postpartum depression in the combined models were mostly distinct from HRV indices predicting postpartum anxiety.

### Pregnancy HRV and symptoms of postpartum depression

HRV indices included as predictors of symptoms of postpartum depression in the combined model reflected alterations in both parasympathetic and sympathetic activity within the ANS. Four of the five significant HRV indices predictive in the depression outcome (Total power baseline, Total power after stressor, LF power baseline, and SDNN after stressor) represent both parasympathetic and sympathetic activity with only baseline measurement of HF power indicative of primarily parasympathetic activity. Unlike the recent study by Singh Solorzano and colleagues [35], RMSSD was not a significant predictor of symptoms of postpartum depression. However, Singh Solorzano and colleagues [35] measured only RMSSD, which is indicative of parasympathetic activity; in the current study, changes in HF power related to parasympathetic activity may reflect similar alterations in the depression outcome group. Decreased HF power and SDNN correspond to results from previous HRV studies in non-pregnant samples [42]. Greater alterations in the balance between the parasympathetic and sympathetic may reflect the inability of this system to respond appropriately to changes in emotional or environmental stimuli. Additionally, the majority of HRV indices predictive of symptoms of postpartum depression in the combined model were from the baseline measurement preceding the stressor, strongly suggesting that HRV alterations during resting-state have predictive value for depressive symptoms.

### Pregnancy HRV and symptoms of postpartum anxiety

With the exception of SDNN after stressor measurement, HRV indices predictive for the anxiety outcome in the combined model differed from those predictive in the depression outcome. Higher RMSSD baseline, lower RMSSD after stressor, and lower HF power after stressor added to the model predicting postpartum anxiety and are thought to be more reflective of parasympathetic activity. These results could suggest a general dysregulation within the parasympathetic nervous system and potential alterations in the adaptive response reflecting an inability to appropriately dampen sympathetic activity. Lower HF power in our combined anxiety outcome model is consistent with results from a previous study by Kimmel and colleagues [8] showing lower HF power in individuals diagnosed with obsessive-compulsive disorder (OCD); albeit no longer considered an anxiety disorder, OCD in the perinatal period is often still associated with anxiety. In contrast to the previous study, we did not find LF power or LF/HF ratio significant in our anxiety prediction model. The previous study focused on investigating how HRV indices measured in the third trimester related to psychiatric history and symptoms in pregnancy; as the current study is utilizing HRV indices in the third trimester to predict depressive or anxiety symptoms postpartum, this may account for the differences. Another HRV study conducted by Braeken and colleagues [49] was in agreement with our study in finding that pregnant women previously diagnosed with an anxiety disorder had lower RMSSD and HF power. In this study, and in contrast to the depression model, three out of four late pregnancy HRV indices predictive of symptoms of postpartum anxiety were derived from the measurement after the stressor. This result could suggest that postpartum depression is more related to baseline ANS activity, whereas anxiety is more related to difficulty recovering from mild stressors. A recent study showed that women with and without anxiety in late pregnancy display differences in the degree of autonomic rebound as indicated by HRV following a stressor [50].

### Self-report measures and symptoms of postpartum depression or anxiety

Higher depression and anxiety scores at gestational week 32 were included in the combined models predicting postpartum symptoms of depression and postpartum symptoms of anxiety; these results were in agreement with previous research in this area [26] and reflective of a general comorbidity between psychological symptoms of depression and anxiety [74]. As previously shown in other studies, lower resilience was predictive of symptoms of PPD [75]. Fear of childbirth at gestational week 32 was predictive of postpartum anxiety in the combined model, which has been previously shown in a study by Jokić-Begić and colleagues [76]; their study showed anxiety sensitivity, defined as the tendency to fear anxiety-related symptoms, indicated a vulnerability factor for fear of childbirth [76]. Finally, use of SSRIs, typically utilized as a treatment method for both depressive and anxiety disorders, was predictive of postpartum anxiety in our combined model. This may be due to comorbidity, with SSRI use reflective of severity of past psychiatric history.

### Limitations

While this study is relatively large for an HRV study, the sample size is relatively small for a predictive study. Despite the number of women presenting with depression and anxiety symptoms postpartum being comparable to the expected percentage in the general population (23.3% for depression and 11.3% for anxiety), the absolute number of women with these symptoms is still low. Further, even fewer women developed de-novo symptoms, making it difficult to delineate the influence of concurrent symptoms on HRV indices. It may be the case that HRV indices were selected in our combined models due to their associations with symptoms of depression and anxiety instead of for their predictive utility. Another limitation is that HRV indices were measured via PPG instead of electrocardiogram (ECG), the gold standard for HRV index acquisition [77]. Previous studies have, however, shown good agreement between HRV measures derived from ECG and PPG [52, 53], particularly when the PPG signal is acquired from the finger as was the case in this study [78]. Still, there is evidence to suggest that PPG-HRV is more sensitive to changes in activity or mental stress than ECG-HRV, which could ultimately impair the agreement between these two measurements [77]. Further studies comparing HRV acquisition using both measures are warranted. Further, the DST, a working memory task, was considered a slight stressor in our study protocol, though there was no data available to verify that the task elicited a stress response; the second HRV measurement may, therefore, not reflect the expected HRV adaptations to a stress reaction. Another limitation is that the depression and anxiety measures (EPDS and BAI, respectively) were conducted during pregnancy week 32, while our HRV indices were measured during pregnancy week 38. Further, the EPDS does not represent depression only; some questions are attributed to anxiety, and the results may have been different using clinical diagnosis as the outcome measure. Another limitation is that the time-point for measurement in late pregnancy excludes women with delivery before 38 weeks, which might represent women with other complications.

### Future directions

Future prediction studies in this area could aim to include a larger sample size or to include only pregnant women with no symptoms of depression or anxiety who later develop these symptoms during the postpartum period. While depression and anxiety measurements during pregnancy are certainly dependable predictors of postpartum outcomes, biological predictors, such as HRV, might prove particularly beneficial for identifying women as high-risk for postpartum depression or anxiety who would, otherwise, have no known clinical risk factors during pregnancy. Future studies utilizing two HRV measurements, one at baseline and one after a stressor, could verify that their task elicits a measurable stress response via, for example, skin conductance response. Future studies could also investigate if there are physiological differences in HRV patterns for women who are non-depressed/non-anxious in pregnancy, but later develop symptoms of postpartum depression and anxiety. In general, studies within this area often group symptoms of depression and anxiety together, but future studies could also aim to further disentangle this relationship to better optimize prediction.

Finally, future studies could investigate if HRV biofeedback (HRVB) helps to improve depressive and anxious symptomatology in perinatal women. HRVB is a type of non-invasive therapy in which real-time information about physiological activity is relayed back to the patient, and the patient responds by making subtle adjustments to reach the target activity level [79]. In HRVB intervention studies, researchers found that HRVB significantly alleviated symptoms of perinatal affective disorders in general, and was particularly beneficial in alleviating anxiety [79, 80]. Further, a meta-analysis consisting of 14 randomized-controlled studies found that HRVB improved depressive symptoms in adults [45]. HRVB could potentially provide a safe, non-pharmacological treatment option for women in the perinatal period.

## Conclusion

To our knowledge, this is the first study to examine the ability of multiple pregnancy HRV indices to predict the development of postpartum anxiety and depression. We found that HRV indices alone were not predictive of symptoms of postpartum depression or anxiety. HRV indices were selected as significant predictors in our combined models, but the addition of HRV indices did not improve already strong prediction models based on psychological variables in the current population where a large proportion of women had symptoms of depression and anxiety during pregnancy. Even so, the combined models included mostly distinct HRV indices as predictive of postpartum symptoms of depression and anxiety, encouraging further investigation into differences in HRV related to symptoms of depression and anxiety. The current study does not provide evidence for the use of HRV indices for prediction of postpartum depression and anxiety in women with known pregnancy depression and anxiety. Further studies investigating the ability of HRV to predict postpartum affective disorders are warranted among women without pregnancy symptoms of depression or anxiety.

### Supplementary information

Supplementary Information

Supplementary Figure 1

Supplementary Figure 2

## Data Availability

The data utilized in this study are available upon reasonable request. Due to privacy and ethical considerations, the data are not publicly available.

## Code availability

Computer code used for this project is shared at: https://github.com/allisoneriksson/Pregnancy_HRV_to_predict_PPD_Anxiety.
