# Supplementary material for: Investigating heart rate variability measures during pregnancy as predictors of postpartum depression and anxiety: an exploratory study
Source: Transl Psychiatry. 2024 May 14;14:203. doi: 10.1038/s41398-024-02909-9 (PMC11094065; doi:10.1038/s41398-024-02909-9)
Supplement: Supplementary file 1 — Supplementary Information [file 41398_2024_2909_MOESM1_ESM.docx]

**Supplementary Figure 1**

*AUC and Significant Predictors from ROC Analysis with Elastic Net Logistic Regression for Model with Depression Outcome Excluding HRV Indices*

*Note.* Depression outcome based on EPDS score ≥ 11; Resilience = total RS-14 score; Depression w. 32 = total EPDS; Anxiety w.32 = total BAI; Model parameters: α = .35 and λ = 0.24

**Supplementary Figure 2**

*AUC and Significant Predictors from ROC Analysis with Elastic Net Logistic Regression for Model with Anxiety Outcome Excluding HRV Indices*

*Note*. Anxiety outcome based on BAI score ≥ 16; Model parameters: α = .75 and λ = 0.17
