# Supplementary figures and images for: Investigating heart rate variability measures during pregnancy as predictors of postpartum depression and anxiety: an exploratory study
Source: Transl Psychiatry. 2024 May 14;14:203. doi: 10.1038/s41398-024-02909-9 (PMC11094065; doi:10.1038/s41398-024-02909-9)

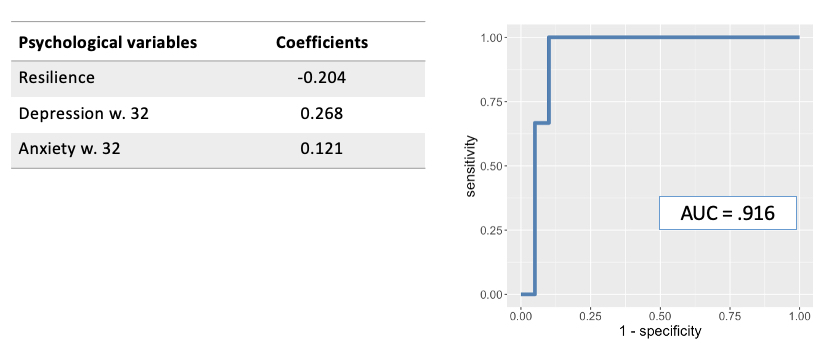

Supplement: Supplementary file 2 — Supplementary Figure 1 [file 41398_2024_2909_MOESM2_ESM.jpg]

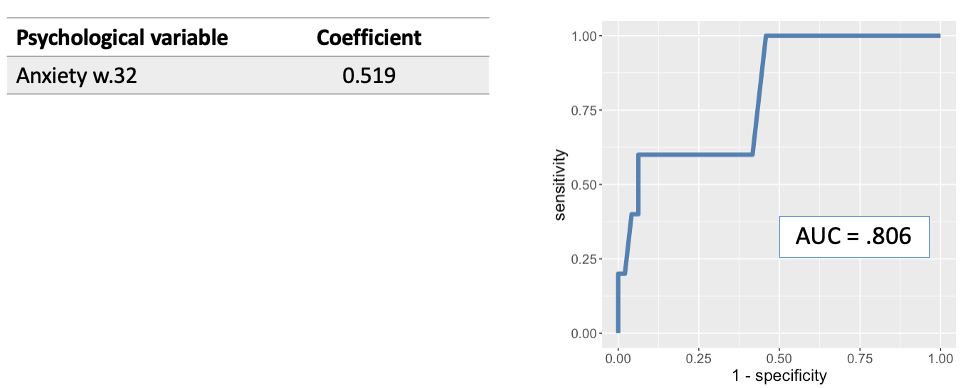

Supplement: Supplementary file 3 — Supplementary Figure 2 [file 41398_2024_2909_MOESM3_ESM.jpg]
